# Behaviors, hygiene habits, and sources of care among removable complete and partial dentures wearers: A multicenter cross‐sectional study

**DOI:** 10.1002/cre2.867

**Published:** 2024-03-03

**Authors:** Radwan Algabri, Ahmed Yaseen Alqutaibi, Sadeq Altayyar, Abdulkarem Mohammed, Ghadeer Khoshafa, Emad Alryashi, Shaher Al‐Shaher, Baghdad Hassan, Gubran Hassan, Motaher Dammag, Sami Al‐Aqab, Shaima Al‐Shami, Abdulrhman Al‐Barakani

**Affiliations:** ^1^ Department of Prosthodontics, Faculty of Dentistry Ibb University Ibb Yemen; ^2^ Department of Prosthodontics, Faculty of Dentistry National University Ibb Yemen; ^3^ Substitutive Dental Science Department, College of Dentistry Taibah University Al Madinah Saudi Arabia; ^4^ Department of Oral Medicine and Periodontology, Faculty of Dentistry Dhamar University Thamar Yemen; ^5^ Department of Prosthodontics, Faculty of Dentistry National University Taiz Yemen

**Keywords:** denture care knowledge, denture hygiene practices, denture maintenance, removable denture wearers

## Abstract

**Objectives:**

There is a lack of data regarding the hygiene practices and sources of care among individuals in Yemen who wear removable complete and partial dentures. The purpose of this study was to explore the behaviors, hygiene habits, and sources of care information among patients who utilize complete and partial dentures in Yemen.

**Materials and Methods:**

A descriptive cross‐sectional research design was utilized, and a sample of 217 consecutive participants who wore removable complete and/or partial dentures were enlisted. A questionnaire was employed to collect data on demographic information, educational attainment, denture habits, denture cleaning practices, and encountered difficulties. Statistical analysis was conducted using SPSS software, and significance was determined using chi‐square tests, with a significance level of .05.

**Results:**

The data analysis revealed that a majority of the participants were male (72.4%) and had an average age of 65.14 years. Fifty‐three percent of the participants wore partial dentures, while 34.6% wore complete dentures. Only 6.5% of the participants wore both complete and partial dentures, and 6% wore overdentures. The majority of participants (88.47%) had dentures made of acrylic material. Additionally, 43% of participants wore dentures while sleeping, and 61.3% stored their dentures in dry places without a water‐filled container when not in use. The most commonly reported cleaning methods were water only (24.4%), followed by water and soap (19.4%). Furthermore, a large portion of the participants (59.4%) received denture care information from dentists. However, the majority (59%) did not visit a dentist for regular denture maintenance or any denture‐related issues. The data analysis did not reveal any significant association between age or education level and denture cleaning methods.

**Conclusion:**

This cross‐sectional survey provides insight into the hygiene knowledge and practices of removable denture wearers in Yemen. The findings underscore the necessity for enhanced oral hygiene education and awareness within this population. The study offers valuable insights for oral health professionals to design targeted interventions and educational initiatives aimed at promoting proper denture care and maintenance. These efforts have the potential to enhance the oral health and overall well‐being of removable denture wearers.

## 1 INTRODUCTION

Oral health is an indispensable human right that empowers individuals to achieve a higher standard of living (Petersen & Kwan, 2010). The prioritization of addressing oral and non‐communicable diseases should be acknowledged as imperative in global public health initiatives (Peres et al., 2019; Tomar & Cohen, 2010). Additionally, there is a growing interest in exploring the connection between oral and general health, as well as identifying oral health indicators that possess significant prognostic or diagnostic value for general health deterioration (Jain et al., 2023; Romandini et al., 2021). The aging of the population has emerged as a notable public health concern due to its association with increased rates of illness, institutionalization, and mortality. These factors can have a direct impact on the health‐related quality of life of older individuals (Damayanthi et al., 2018).

Rehabilitating individuals with partial or complete edentulism encompasses a variety of treatment options, each presenting distinct advantages and disadvantages (Peracini et al., 2010). Dentures, composed of a denture base (constructed from materials such as acrylic, metal alloys, or polymers) and denture teeth (typically made from acrylic, composite resin, or porcelain), are commonly utilized. However, despite their smooth surfaces, denture plaque can still accumulate in the “tooth–gingivae” interface (Ogunrinde & Opeodu, 2015; Shankar et al., 2017).

Poor oral hygiene can lead to various negative consequences such as pain, challenges in eating and speaking, and broader health problems (Petersen et al., 2005). To tackle these issues, Oral Health Promotion (OHP) places its emphasis on increasing knowledge and understanding, delivering educational programs, and implementing preventive measures to combat periodontal diseases, tooth decay, and oral cancers (Petersen & Kwan, 2010).

Insufficient adherence to oral hygiene practices, particularly the build‐up of dental plaque, plays a significant role in the development of various oral conditions such as dental caries, periodontal diseases, and systemic infections. Furthermore, both periodontal diseases and inadequate oral hygiene in individuals with natural teeth and dentures have been identified as risk factors for aspiration pneumonia among hospitalized patients and residents of nursing homes (Ástvaldsdóttir et al., 2018; Scannapieco, 2023). It is worth noting that wearing dentures during sleep not only leads to oral inflammation and a higher presence of microorganisms but also increases the risk of pneumonia by 2.3 times. These findings underscore the potential importance of implementing oral hygiene programs aimed at preventing pneumonia within the community (Iinuma et al., 2015).

Raising awareness and promoting motivation among individuals who wear removable partial dentures (RPDs) is of paramount importance to foster appropriate oral hygiene practices for their remaining natural teeth. This is crucial for preserving the health and integrity of both the teeth and the surrounding periodontal tissues (Dhingra, 2012). Patients who wear RPDs are inherently more susceptible to developing gingivitis and are also more prone to tooth decay. The maintenance of good oral hygiene plays a pivotal role in the overall dental health of these patients, as it directly impacts the condition of the remaining teeth and the effectiveness of retaining the removable appliances (Lamfon, 2023).

Studies have shown that older adults frequently demonstrate inadequate denture cleaning practices and lack appropriate oral hygiene habits (Al‐Hadi Hamasha et al., 2000; Esan et al., 2004; Tramini et al., 2007; Vyšniauskaité et al., 2005). Several factors contribute to this phenomenon, including social status, age, education, systemic diseases, and smoking. Moreover, a lack of knowledge regarding oral healthcare maintenance and infrequent dental check‐ups exacerbate this problem (Cankaya et al., 2020).

In a study conducted by Ambikathanaya et al (Ambikathanaya et al., 2022), the researchers examined the prevalence of dental caries among individuals wearing acrylic RPDs with and without diabetes. The findings indicated that the presence of an RPD had a minimal impact on the occurrence of dental caries when participants practiced good oral hygiene, followed postinsertion RPD instructions, and maintained regular dental visits, regardless of their diabetic status. Another study, conducted by Lamfon (Lamfon, 2023), investigated the impact of a reinforced audiovisual oral health educational aid on the oral health of patients with RPDs. The findings revealed that reinforced oral health education effectively enhanced adherence to oral hygiene measures, reduced plaque accumulation, and improved gingival health among the participants.

In a study conducted by Patel et al. (Patel et al., 2012), the researchers analyzed the behaviors and hygiene habits of complete denture patients within the local population of Ahmedabad. The findings of the study indicated that the surveyed edentulous patients exhibited a satisfactory frequency of cleaning both their dentures and oral cavity. Additionally, it was observed that these patients had a habit of removing their dentures at night. Nevertheless, the study revealed that the methods and products employed for denture care were deemed insufficient.

Two primary approaches are advocated for the eradication of material from dentures: mechanical and chemical methods. Mechanical methods encompass brushing with water, soap, dentifrice, or abrasives, as well as ultrasonic treatment. Chemical methods can be categorized according to their composition and mode of action, which includes hypochlorides, peroxides, enzymes, acids, crude drugs, and mouthwashes (oral rinses) specially formulated for denture cleansing (Nikawa et al., 1999).

A review conducted by McReynolds et al. (2023) examined the topic of denture stomatitis and emphasized the significance of implementing sustained behavioral changes for achieving effective treatment outcomes. The authors also underscored the limited advantages of long‐term usage of antifungal medications and highlighted the role of managing denture stomatitis in mitigating the likelihood of pneumonia and associated mortality. Furthermore, a separate study conducted by Cankaya et al. (Turgut Cankaya et al., 2020) discovered that denture cleanliness was influenced by the type of advice received and oral hygiene habits, thus underscoring the importance of patient education and motivation in denture maintenance, particularly among geriatric patients.

There is a lack of data regarding the hygiene habit and sources of care among individuals in Yemen who wear removable complete and partial denture. This study aimed to explore the behaviors, hygiene habits, and the sources of care information of patients wearing complete and/or partial dentures in the local populations of Ibb and Taiz cities in Yemen.

## 2 MATERIALS AND METHODS

This descriptive cross‐sectional study was conducted at Ibb University, National University‐Ibb branch, and National University‐ Taiz branch in Yemen. A total of 217 consecutive participants wearing removable complete and/or partial dentures were enrolled in the study. The participants were randomly recruited over a period of 8 months, from March 1, 2023, to October 30, 2023. Before their inclusion in the study, the patients were provided with comprehensive information about the research, and each patient voluntarily provided written consent after being fully informed. The study was conducted with the approval of the Ethical committee at College of Dentistry, Taibah University, Al Madinah, Saudi Arabia.

The study's inclusion criteria encompassed male and female participants aged 18 years and above, who had been utilizing removable complete and/or partial dentures for a minimum of 6 months. Furthermore, the individuals were mandated to demonstrate a willingness to participate in the study and possess sound physical and mental well‐being. To facilitate convenience for the participants, a well‐designed questionnaire was administered in Arabic. This questionnaire was specifically devised by the researchers to evaluate the knowledge and attitudes of denture wearers with respect to denture care and hygiene. Its primary objective was to gather information and gain insights from the participants regarding these particular aspects.

A pilot study was performed involving 10 individuals to assess the questionnaire and guarantee uniform interpretation. It took approximately 15–20 min to complete the questionnaire. The data gathered during the pilot study were not incorporated into the primary study. The sample size for the primary study was determined to be 217, based on an 85% response rate in the initial study, a 95% confidence interval, and a desired absolute precision of 3%. The overall sample size was calculated using Milward (Milward et al., 2013) and Ogunrinde (Ogunrinde & Opeodu, 2015).

The questionnaire utilized in this study encompassed various subjects pertaining to the characteristics of the participants, including demographic information, educational background, and habits such as smoking. It also obtained information about the types and quantity of dentures utilized, as well as the materials employed in their fabrication and the duration of denture use. The questionnaire further delved into factors such as whether the participants wore their dentures while sleeping, how they stored their dentures when not in use, whether they cleaned their dentures inside or outside of their mouth, the frequency of denture and natural teeth cleaning per day, and any challenges encountered in cleaning their dentures. Additionally, the questionnaire included inquiries regarding the methods, devices, and materials utilized for denture cleaning. Furthermore, it assessed the concerns of the participants regarding their previous dental visits and whether their previous dentist had provided instructions on denture cleaning. The data was collected and analyzed using the SPSS software. The Chi‐square test was employed to assess the statistical significance between the variables, and the level of significance was set at *p* < .05.

## 3 RESULTS

During the 8‐month audit period, a total of 217 participants successfully completed the questionnaires. The data revealed that 72.4% of the participants were male, while 27.6% were female. The age range of the participants varied from 30 to 100 years old, with an average age of 65.14. Specifically, 55.3% of the participants fell within the age range of 51−70 years old. Further analysis of the data indicated that 77% of the participants had not received any formal education, while 9.2% had obtained a university education. Additionally, 13.8% had completed their high school education. Lastly, 38.7% of the participants reported being smokers, as presented in Table 1.

**Table 1:** Distribution of participants according to their demographical characteristics and habits (*n* = 217).

Characteristics		Frequency (*n*)	Percentage (%)
Gender	Male	157	72.4
	Female	60	27.6
	**Total**	**217**	**100**
Age groups	30–50 years	50	23
	51–70 years	120	55.3
	More than 71 years	47	21.7
	**Total**	**217**	**100**
Education level	University	20	9.2
	High school	30	13.8
	Uneducated	167	77
	**Total**	**217**	**100**
Smoking	Yes	84	38.7
	No	133	61.3
	**Total**	**217**	**100.0**

*Note*: Bold values indicate *p* < 0.05.

The participants were divided into different categories based on the type of removable dentures they wore. Of the participants, 53% wore partial dentures, 34.6% wore complete dentures, 6.5% wore a combination of complete dentures and partial dentures, and 6% wore overdentures. It is worth noting that each arch had various combinations of denture types. In terms of material, the majority of participants (88.47%) wore acrylic dentures, while 6.45% wore metallic cobalt chrome dentures and 5.1% wore flexible dentures (Table 2).

**Table 2:** Distribution of participants based on the types and materials of dentures (*n* = 217).

Characteristics		Frequency (*n*)	Percentage (%)
Types of dentures	Partial dentures	115	53
	Complete dentures	75	34.6
	Complete with partial dentures	14	6.5
	Overdentures	13	6
	**Total**	**217**	**100**
Denture types based on arch	Upper partial and lower partial	54	24.9
	Only lower partial	35	16.1
	Only upper partial	26	12
	Upper complete and lower complete	48	22.1
	Only lower complete	14	6.5
	Only upper complete	13	6
	Upper partial and lower complete	7	3.2
	Upper complete and lower partial	7	3.2
	Upper complete overdenture and lower complete overdenture	13	6
	**Total**	**217**	**100**
Denture material types	Acrylic	192	88.47
	Metal	14	6.45
	Flexible	11	5.1
	**Total**	**217**	**100**

*Note*: Bold values indicate *p* < 0.05.

Table 3 presents the participants’ previous denture experiences. The findings indicate that 51.6% of the participants had their current dentures as their initial denture, while 28.1% had worn two dentures. Additionally, 10.6% had worn three dentures, and 9.7% had worn more than three dentures in the past. In terms of duration, 45.2% of the participants had been wearing their current removable partial and/or complete dentures for 6 months to 1 year. Furthermore, 30% had been wearing them for 1−4 years, 13.8% for 5−10 years, and 11.1% for more than 10 years.

**Table 3:** Distribution of participants based on the number of dentures and duration of wearing the denture (*n* = 217).

Characteristics		Frequency (*n*)	Percentage (%)
Number of previous dentures	One denture	112	51.6
	Two dentures	61	28.1
	Three dentures	23	10.6
	More than three dentures	21	9.7
	**Total**	**217**	**100**
Duration of denture usage	6 months to 1 year	98	45.2
	1–4 years ago	65	30
	5–10 years ago	30	13.8
	More than 10 years	24	11.1
	Total	217	100
	**Total**	**217**	**100**

*Note*: Bold values indicate *p* < 0.05.

The data analysis revealed that 43.3% of the participants (94 out of 217) reported wearing their dentures while sleeping. Additionally, 73.7% of participants removed their dentures at some point during the day. Regarding denture storage practices, 61.3% mentioned storing their dentures in different locations without water, while 38.7% stated that they typically store their dentures in a container filled with water (Table 4).

**Table 4:** Distribution of participants based on the habits of sleeping with dentures, daytime denture removal, and denture storage location (*n* = 217).

Characteristics		Frequency (*n*)	Percentage (%)
Sleeping with dentures	Never	123	56.7
	Every night	27	12.4
	Sometimes	43	19.8
	Often	16	7.4
	Rarely	8	3.7
	**Total**	**217**	**100**
Daytime denture removal	Yes	160	73.7
	no	57	26.3
	**Total**	**217**	**100**
Where participants store their dentures when not in use	Inside a container with water	84	38.7
	Desk drawer	11	5.1
	Pocket	54	24.9
	Soft napkin	10	4.6
	In the wallet	3	1.4
	Plastic bag	6	2.8
	Inside empty container	38	17.5
	Never	11	5.1
	**Total**	**217**	**100.0**

*Note*: Bold values indicate *p* < 0.05.

Regarding daily denture cleaning, 66.8% of participants reported cleaning their dentures on a daily basis, while the remaining 33.2% indicated that they do not. Among those who clean their dentures, 46.5% clean them once a day. Other frequencies of cleaning included twice daily (18%), three times a day (7.8%), more than three times a day (4.6%), once weekly (4.6%), and 18.4% of the participants who clean their dentures do not clean them regularly or at any specific frequency throughout the day (Table 5).

**Table 5:** Distribution of participants based on the questions about cleaning of the denture (*n* = 217).

Characteristics		Frequency (*n*)	Percentage (%)
Daily denture cleaning frequency	Yes	145	66.8
	No	72	33.2
	**Total**	**217**	**100**
Denture cleaning frequency	No	40	18.4
	Once daily	101	46.5
	Twice daily	39	18.0
	Three	17	7.8
	More than three	10	4.6
	Once weekly	10	4.6
	**Total**	**217**	**100**
Denture cleansing methods	No	23	10.6
	Toothbrush and soap	29	13.4
	Toothbrush and paste	20	9.2
	Toothbrush and water	8	3.7
	Only water	53	24.4
	Water and soap	42	19.4
	Water and NaOCl	2	0.9
	Messwa'k	12	5.5
	Water with lemon	5	2.3
	Only Toothbrush	18	8.3
	Denture cleanser tabs	5	2.3
	**Total**	**217**	**100.0**
Disinfectant use for denture cleaning	No	177	81.6
	Ethanol	6	2.8
	NaOCl	20	9.2
	Soap and NaOCl	1	0.5
	Normal saline and NaOCl	1	0.5
	Mouth wash	10	4.6
	Dettol	2	.9
	**Total**	**217**	**100.0**
Where participants clean their dentures	No	35	16.1
	Outside	165	76.1
	Inside	17	7.8
	**Total**	**217**	**100**
Challenging areas of denture cleaning	No	84	38.7
	Between teeth	58	26.7
	Denture border	6	2.8
	The denture border and between teeth	3	1.4
	Behind teeth	1	0.5
	Palatal surface	4	1.8
	Tissue side	46	21.2
	Between teeth and tissue side	11	5.1
	Occlusal surfaces	4	1.8
	**Total**	**217**	**100.0**

*Note*: Bold values indicate *p* < 0.05.

Common methods of cleaning included using water only (24.4%), water and soap (19.4%), toothbrush and toothpaste (9.2%), toothbrush and soap (13.4%). 10.6% of participants did not use any specific method. Disinfectants were used by a small minority, with sodium hypochlorite (9.2%) and mouth wash (4.6%) being the most common choices. The majority (76.1%) cleaned their dentures outside the mouth. When asked about challenging areas to clean, 26.7% of participants mentioned the spaces between denture teeth, 21.2% mentioned the tissue side of the denture, and 38.7% found no difficult areas (Table 5).

In relation to regular dental check‐ups, a majority of participants (59%) did not seek a dentist's assistance for the maintenance of their dentures or any issues related to dentures. Approximately 33.7% of participants only visited a dentist when they encountered a problem with their dentures. A small percentage (4.1% and 3.2%) visited the dentist every 6 months and once a year, respectively. As for the sources of information on denture cleaning, the majority (59.4%) received information from dentists, 18.9% received it from friends or relatives, 5.5% obtained it from the Internet or other sources, and 8.3% reported not receiving any information. Regarding participants' responses on previous instructions from dentists regarding denture cleaning, 70% stated that they received instructions, 11.1% did not receive any, and 18.9% could not recall (Table 6).

**Table 6:** Distribution of participants based on the dental check‐ups, cleaning information sources, and dentist's instructions (*n* = 217).

Characteristics		Frequency (*n*)	Percentage (%)
Regular dental check‐ups	No	128	59.0
	Yes, every 6 months	9	4.1
	Yes, once a year	7	3.2
	Yes, when I have a problem	73	33.7
	**Total**	**217**	**100.0**
Sources of denture cleaning information	There is no any information	18	8.3
	Internet	12	5.5
	Dentist	129	59.4
	Friend	8	3.7
	Friend or relative	41	18.9
	Self	5	2.3
	TV	2	0.9
	Dentist and friend and relative	2	0.9
	**Total**	**217**	**100**
Previous dentist's instructions on denture cleaning	Yes	152	70.0
	No	24	11.1
	I don't remember	41	18.9
	**Total**	**217**	**100**

*Note*: Bold values indicate *p* < 0.05.

Among the 140 participants who had remaining natural teeth supporting partial dentures or overdentures, it was found that 51.5% only cleaned the denture, while 36.4% cleaned both the denture and their remaining natural teeth. Additionally, 12.1% of the participants did not clean either. Looking specifically at the brushing frequency for the remaining natural teeth, it was observed that 58.6% brushed once daily, 8.6% brushed twice daily, 6.4% brushed three times, 5% brushed more than three times, 4.3% brushed once weekly, 5% brushed once monthly, and 12.1% did not perform any cleaning. These findings are summarized in Table 7.

**Table 7:** Distribution of participants' cleaning habits for remaining natural teeth (*n* = 140) with partial dentures, overdentures, and complete dentures with opposing partials.

Characteristics		Frequency (*n*)	Percentage (%)
Denture cleaning approach (natural teeth included or dentures only)	No	17	12.1
	With the remaining natural teeth	51	36.4
	The denture only	72	51.5
	**Total**	**140**	**100**
Frequency of brushing remaining natural teeth per day	No	17	12.1
	Once daily	82	58.6
	Twice daily	12	8.6
	Three	9	6.4
	More than three	7	5.0
	Once monthly	7	5.0
	Once weekly	6	4.3
	**Total**	**140**	**100**

*Note*: Bold values indicate *p* < 0.05.

**Figure 1:**
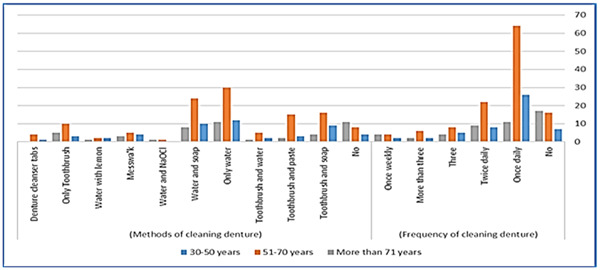
Bar chart illustrates the correlation between patient's age and the frequency and methods used for denture cleaning.

**Figure 2:**
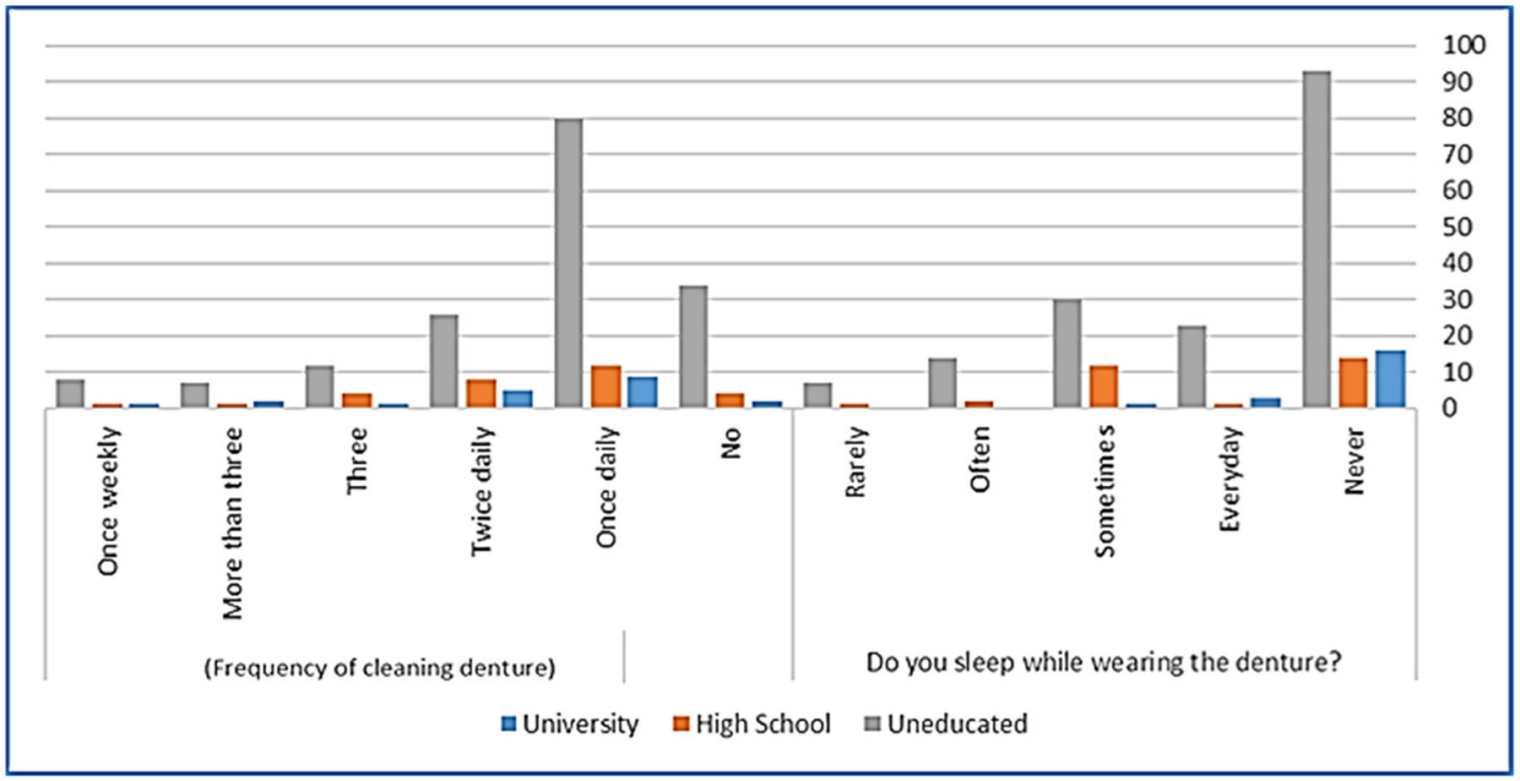
Bar chart depicts relationship between patients' education level, frequency of denture cleaning, and overnight wearing.

**Figure 3:**
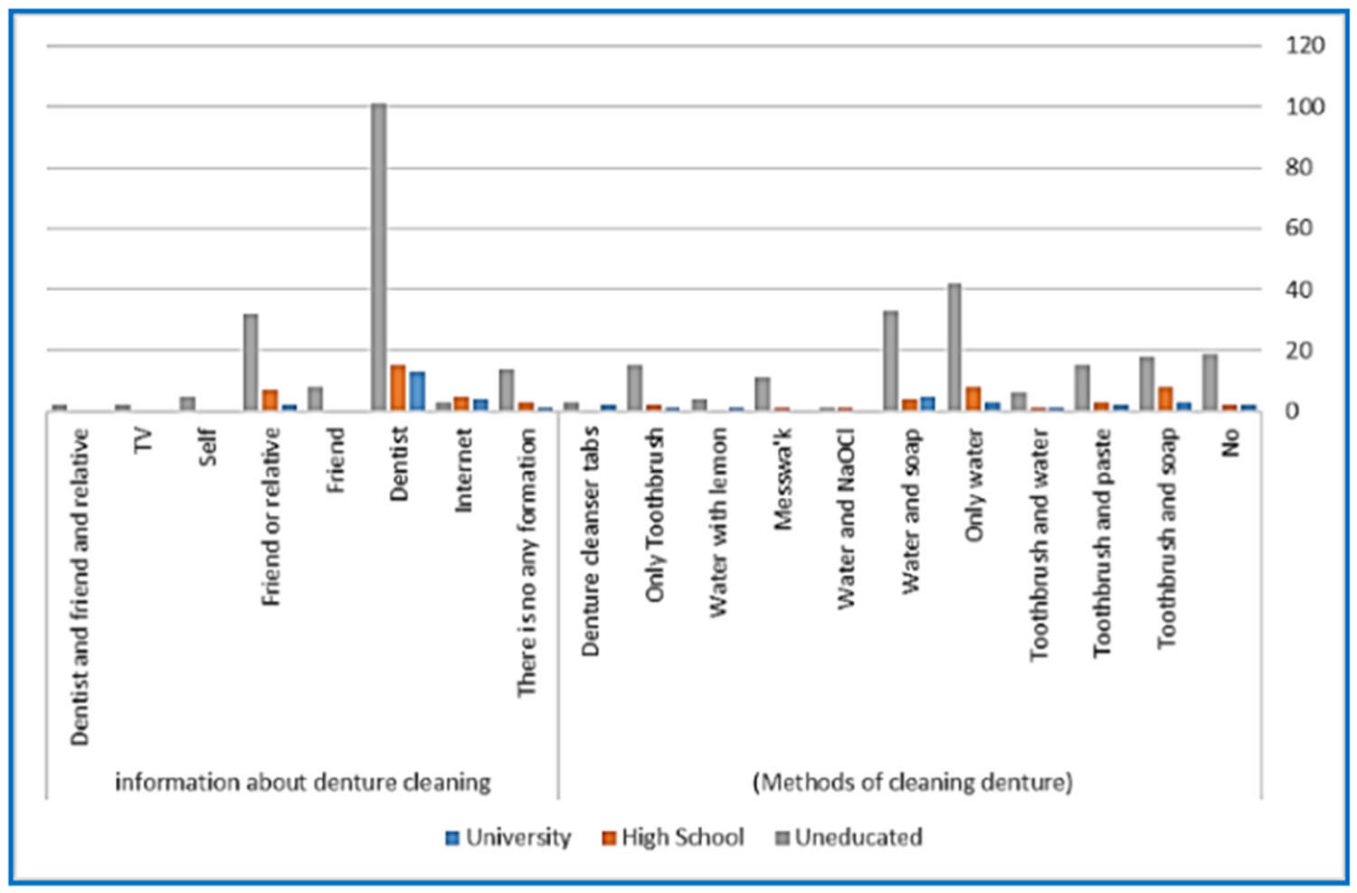
Bar chart represents the association between the education level of patients and the methods and sources of information they utilize for denture cleaning.

There was no significant association between the age of participants and their denture cleaning methods. However, a statistically significant association was found between cleaning dentures at least once a day and the age range of 51−70 years old (*p* = .030) (Figure 1). Similarly, the methods and frequency of denture cleaning did not significantly correlate with participants' education level, except for uneducated participants who were more likely to sleep with dentures overnight (*p* = .041) (Figure 2). Additionally, a significant statistical association was observed between education level and sources of denture cleaning information. Uneducated participants relied more on these sources compared to educated participants (*p* < .05) (Figure 3).

## 4 DISCUSSION

This is the first cross‐sectional study conducted in Yemen that examines the oral hygiene knowledge of individuals who wear removable dentures. The study aimed to evaluate their awareness and comprehension of oral care practices and denture maintenance.

It is essential for individuals who wear removable dentures to have regular dental visits and practice proper denture hygiene, as this greatly influences the longevity of their dentures and enhances oral health (Peracini et al., 2010). Previous reports have highlighted the lack of adequate instructions provided to patients regarding denture hygiene and care, which further complicates the situation (Dikbas et al., 2006; Patel et al., 2012; Saha et al., 2014).

In the present study, a significant proportion (72.4%) of the participants was male, whereas only 27.6% were female. This disparity in gender distribution can be attributed to prevailing cultural norms and traditional gender roles in Yemen. These societal expectations and responsibilities may restrict women's involvement in research studies, as well as limit their mobility. Moreover, the majority (88.47%) of the participants in our study utilized acrylic removable dentures, while smaller proportions (6.45% and 5.1%) wore metallic and flexible removable dentures, respectively. This discrepancy may be due to factors such as the cost, lower income levels, and limited availability of dental technicians specializing in these denture types in Yemen. Additionally, variations in educational levels, socioeconomic statuses, and restricted access to dental care could also contribute to this issue.

The study discovered that a majority of participants (51.6%) utilized a solitary denture, with shorter durations of usage in comparison to prior research. It is plausible that factors such as educational attainment, socioeconomic status, and limited access to dental care may contribute to this outcome (Compagnoni et al., 2007; Peracini et al., 2010). It is worth noting that removing dentures at night is advisable to prevent stomatitis induced by microorganisms, particularly those belonging to the Candida species (de Castellucci Barbosa et al., 2008). Surprisingly, 43.3% of participants wore dentures while sleeping, whereas 56.7% took them off overnight. These findings are in line with similar investigations, (de Castellucci Barbosa et al., 2008; Peracini et al., 2010) yet diverge from others wherein lower percentages of participants were reported to engage in the practice of sleeping with dentures (Johnson, 2013; Ogunrinde & Opeodu, 2015). Prolonged use of complete dentures was found to be more frequently observed among patients diagnosed with denture stomatitis, a pathological condition associated with inflammation of the oral tissues (Baran & Nalçacı, 2009; Çakan et al., 2015; Raab et al., 1991; Takamiya et al., 2011).

In our study, it was discovered that 24.4% of participants engaged in the practice of cleaning their dentures using tap water, a result that aligns with the findings of previous studies (Aoun & Gerges, 2017; Apratim et al., 2013). Furthermore, it was revealed that merely 38.7% of participants stored their dentures in containers filled with tap water. This findings is noteworthy in light of the 2011 guidelines provided by the American College of Prosthodontists, which recommend the storage of dentures in water both after cleaning and during periods of non‐use to prevent the risk of warping (Felton et al., 2011).

Our study found that 34.6% of participants utilized brushing as a mechanical cleaning technique, a result consistent with previous studies (Dikbas et al., 2006; Kulak‐Ozkan et al., 2002). However, our finding is comparatively lower in relation to other studies (Marchini et al., 2004; Ogunrinde & Opeodu, 2015; Patel et al., 2012; Peracini et al., 2010), potentially due to individuals continuing their pre‐denture hygiene practices. The popularity of brushing can be attributed to its accessibility, simplicity, and affordability (Ferruzzi et al., 2015). Merely 2.3% of the participants reported using denture cleansers, which could be related to potential insufficient guidance from dentists, the higher cost associated with denture cleansers, and the limited availability of these products in the market.

Peracini et al. (2010) conducted a study which indicated that cleaning certain areas of complete dentures poses a challenge, specifically the internal labial flange, inner surface, and acrylic teeth. Our study findings revealed that a notable percentage of participants (26.7% and 21.2%) identified the spaces between artificial teeth and the inner surface of the denture base, respectively, as the most challenging areas to clean. Moreover, 13.4% of participants reported difficulties in cleaning various regions of the denture, including the denture border, palatal surfaces, and occlusal surfaces of denture teeth. These findings highlight the significance of providing explicit instructions for cleaning these specific components of removable dentures.

This study sheds light on the insufficient adherence to routine dental check‐ups among the participants, thus underscoring the significance of regular dental visits in the context of effective denture treatment. Surprisingly, a mere 4.1% of patients reported visiting the dentist every 6 months, while an astonishingly low 3.2% visited once a year. Furthermore, an alarming 59% of participants did not seek dental care subsequent to receiving their dentures, with 33.7% only visiting the dentist when necessitating treatment. This prevailing pattern is in line with earlier research findings and can be attributed to various factors, including limited family incomes, higher costs associated with private dental services, and the absence of government‐funded dental care in Yemen.

Konstantopoulou and Kossioni (Konstantopoulou & Kossioni, 2023) conducted a study on the sources of oral hygiene information among older adults in urban areas. The findings of the study showed a positive association between adherence to oral hygiene practices and receiving guidance from dentists. However, it was observed that only a small number of participants actually remembered receiving oral health advice from dentists. To enhance communication with older patients, the study suggested that dentists should utilize more effective educational strategies, such as digital technology, and provide oral health education to non‐dental healthcare professionals who have interactions with older adults.

Another study conducted by Ferruzzi et al. (Ferruzzi et al., 2015) discovered that 70% of the participants received verbal instructions from dentists on the topic of denture cleaning. Furthermore, Dwivedi et al. (Dwivedi et al., 2021) highlighted the effectiveness of online denture cleansing education sessions in increasing the frequency of daily denture cleaning.

Dental general practitioners exhibited limited knowledge and attitudes regarding patient education in the context of denture care, whereas specialists demonstrated sufficient knowledge, attitudes, and practices regarding denture hygiene and patient education in denture care (Elhddad et al., 2023). Our study examined the association between patients' educational attainment and the practice of wearing dentures overnight. It was found that individuals with lower levels of education were significantly more likely to sleep with their dentures. These findings contrast with those of a prior investigation conducted by Akar and Ergül (Akar & Ergül, 2008), which did not identify a statistically significant connection between educational background and the habit of wearing dentures overnight.

The outcomes of this study concerning the hygiene awareness of individuals who wear removable dentures in Yemen hold a multitude of prospective applications. Primarily, the discoveries from this research may be employed to inform and direct oral health education initiatives customized specifically for this particular population. By comprehending the deficiencies in knowledge and the difficulties faced by denture wearers, oral health practitioners can devise precise interventions aimed at enhancing oral hygiene habits and advocating for superior denture maintenance. Such programs may emphasize the significance of appropriate denture cleansing techniques, storage practices, and regular dental examinations, thereby resulting in enhanced oral health consequences.

Second, the findings can be utilized to formulate guidelines and recommendations for denture care and maintenance in Yemen. This involves identifying the prevailing types of dentures, cleaning techniques, and challenges encountered by denture wearers. Healthcare professionals can establish standardized protocols for denture cleaning and maintenance. These guidelines aim to guarantee that denture wearers receive consistent and evidence‐based guidance on proficiently cleaning and caring for their dentures, thereby minimizing the likelihood of oral health problems and systemic infections associated with inadequate denture hygiene.

Lastly, the findings of this study have broad implications for healthcare policy and resource allocation, particularly in the field of oral health. It is imperative for healthcare policymakers to have a comprehensive understanding of the hygiene knowledge and practices among individuals who wear removable dentures. Such knowledge can empower policymakers to make informed decisions relating to the provision of oral health services. In light of the study's results, policymakers can use this information to develop policies that prioritize oral health education, increase accessibility to dental care, and promote the availability of affordable denture cleaning products. By addressing the specific needs of denture wearers, policymakers can effectively work toward enhancing the overall oral health outcomes of this population and alleviating the burden of oral diseases in Yemen.

While this cross‐sectional survey provides valuable insights into the hygiene knowledge of removable denture wearers in Yemen, it is important to acknowledge certain limitations. The study sample was obtained from specific multicenter locations in Ibb and Taiz cities, which may not be representative of the entire Yemeni population. This sampling approach could introduce selection bias and restrict the generalizability of the findings to other regions or populations within Yemen. Our study did not examine the impact of socioeconomic factors on denture care practices, which can significantly influence individuals' access to dental care, affordability of denture cleaning products, and oral health education. Future research should address this gap to obtain a more comprehensive understanding of denture care practices.

The data collected in this study relied on self‐reported information provided by the participants, which may introduce the possibility of recall bias and social desirability bias. Participants may not accurately remember or report their denture habits, or they may provide responses they believe are socially desirable. The reliance on self‐reported data may also result in information bias, affecting the accuracy and reliability of the results.

Furthermore, being a cross‐sectional study, this research design merely captures a momentary glimpse of the participants' knowledge and practices pertaining to hygiene at a particular point in time. It fails to facilitate an assessment of any changes or patterns that may occur over a period of time. Employing a longitudinal design would furnish more substantial evidence concerning the impact of oral health education and interventions on denture care practices and oral health outcomes. Additionally, it is worth noting that this study neglected to include a control group consisting of individuals who do not wear dentures, thus hindering the ability to make a comprehensive comparison. The inclusion of a control group would have enabled a more thorough understanding of the unique hygiene challenges faced by removable denture wearers, in contrast to those who do not wear dentures. Acknowledging these limitations is crucial to accurately interpret the findings of the study, guide future research aimed at addressing these limitations, and enhance our overall understanding of the hygiene knowledge and practices among removable denture wearers in Yemen.

## 5 CONCLUSIONS

Based on the results of this study, the following conclusions can be drawn:

1. Insufficient knowledge and awareness regarding denture care and oral hygiene are contributing factors to inadequate denture maintenance and cleaning practices among elderly individuals.
2. Implementation of comprehensive oral health education programs leads to improved adherence to oral hygiene, reduced plaque accumulation, and enhanced gum health in denture wearers.
3. The cleanliness of dentures is dependent on advice and oral hygiene habits, rather than factors such as smoking, overnight use, or the age of dentures.
4. Patient education and motivation are crucial for successful denture maintenance. It is imperative to emphasize the importance of providing instructions for denture maintenance and storage.
5. Further research is warranted to develop customized oral health education initiatives for removable denture wearers in Yemen, with a focus on promoting proper denture care, oral hygiene practices, and prevention of oral diseases.

## 6 CLINICAL IMPLICATIONS

By understanding the gaps in knowledge and the challenges denture wearers face, oral health professionals can develop targeted interventions to improve oral hygiene practices and promote better denture care.

## AUTHOR CONTRIBUTIONS

**Radhwan Algabri**: Conceptualization, methodology, investigation, results, writing e original draft, writing e review and editing, project administration, supervision. **Ahmed Yaseen Alqutaibi**: Conceptualization, methodology, investigation, results, writing e original draft, writing e review and editing. **Sadeq Altayyar and Abdulkarem Mohammed**: Results, writing e original draft, writing e review, supervision. **Ghadeer Khoshafa, Emad Alryashi, and Shaher Al‐Shaher**: Methodology, investigation, results. **Baghdad Hassan, Gubran Hassan, Motaher Dammag, and Sami Al‐Aqab**: Investigation, results, project administration. **Shaima Al‐Shami and Abdulrhman Al‐Barakani**: Methodology, investigation, results.

## CONFLICT OF INTEREST STATEMENT

The authors declare no conflict of interest.

## ETHICS STATEMENT

The research protocol received approval from the Faculty of Dentistry ethics committee at Taibah University, with the assigned reference number #111123 and each patient voluntarily provided written consent after being fully informed.

## Data Availability

The data that support the findings of this study are available from the corresponding author upon reasonable request.
